# Prevalence and Susceptibility Pattern of Multi Drug Resistant Clinical Isolates of Pseudomonas aeruginosa in Karachi

**DOI:** 10.12669/pjms.305.5400

**Published:** 2014

**Authors:** Fouzia Khan, Adnan Khan, Shahana Urooj Kazmi

**Affiliations:** 1 Fouzia Khan, MBBS, M.Phil, Department of Microbiology, University of Karachi, Karachi- 75270, Pakistan.; 2 Adnan Khan, PhD, Department of Microbiology, University of Karachi, Karachi- 75270, Pakistan.; 3 Shahana Urooj Kazmi, PhD, Department of Microbiology, University of Karachi, Karachi- 75270, Pakistan.

**Keywords:** *Multi Drug resistant Pseudomonas aeruginosa*, *Amikacin*, *Co-trimaxazole*, *Quinolones*

## Abstract

***Objective:*** To determine the frequency and susceptibility pattern of multi-drug resistant (MDR) *Pseudomonas aeruginosa *isolated from clinical specimens in Karachi.

***Methods:*** This cross sectional study was conducted in Microbiology Department, University of Karachi, from January 2012 to January 2013. Clinical specimens were collected from different hospitals of Karachi. Clinical isolates were identified by standard and specific microbiological methods. The antibiotic susceptibility pattern was determined by Kirby Bauer Disc diffusion method. Clinical and Laboratory Standards Institute (CLSI) guidelines were used to determine the results.

***Results:*** The frequency of MDR *P. aeruginosa* isolated from different clinical specimens was found to be 30%. Amikacin was found to be the most effective antibiotic, followed by Co-trimaxazole and Quinolones.

***Conclusion:*** Antibiotic resistant *P. aeruginosa* are emerging as a critical human health issue. There is an urgent need to resolve the issue by taking some preventive measures. Combined efforts of health care professionals and researchers are required to educate people about the proper use of antibiotics and other infection control measures.

## INTRODUCTION

Antibiotic resistance is one of the alarming issues, affecting human health. There are various factors responsible to the emergence of resistance such as, misuse and overuse of antibiotics, patient related factors, inappropriate prescriptions by the physicians, self medications especially young adults, use of broad spectrum antibiotics and synergistic combinations, un necessary promotions by pharmaceutical industry, untrained staff in microbiological testing laboratories, lack of awareness with the new guidelines recommended for antimicrobial testing etc.^1^ *Pseudomonas aeruginosa* is one of the major cause of hospital acquired infections especially patients admitted in ICU (Intensive Care Unit).^2^ Data presented by the Center for Disease Control and Prevention(CDC), Nosocomial Infection Surveillance System, in the USA, P.aeruginosa cause diverse variety of infections and was found to be the second most common cause of nosocomial pneumonia, the third most common cause of nosocomial urinary tract infections, and the eighth most common cause of nosocomial bacteraemia.^3^ Majority of the infections caused by P. aeruginosa are often severe, life threatening and are un treatable because of the higher resistance to antimicrobial agents and lack of new drugs development.^4^^,^^5^ Over all, resistance rates keep on increasing and differ according to epidemiology of different geographical locations. Multi drug resistance is getting common phenomenon and resistance of almost all anti-pseudomonal agents are being reported worldwide. There is debatable issue of using combination of antimicrobial agents against complicated infections, but usually single antimicrobial agents are recommended for uncomplicated infection.^2^

Development of antimicrobial resistance limits the therapeutic options that leads to high mortality and morbidity.^6^ Emergence of antibiotic resistance in P. aeruginosa has been an increasing trend. There is diversity of definitions to describe MDR isolates of *P. aeruginosa*. According to the different studies, the term MDR *P. aeruginosa* has been described as resistance to at least three antibiotics from a variety of antibiotic classes, mainly Aminoglycosides, Penicillins, Carbapenems, Cephalosporins and Quinolones.^7^ Hidron et al, considered MDR *P. aeruginosa* when resistant to only a single important anti -*P. aeruginosa* agent.^8^

Current study followed the definition of MDR *P. aeruginosa* as stated by European Center for Disease Prevention and Control (ECDC) and Centre for Disease Control and Prevention (CDC), where MDR *P. aeruginosa* was defined as the one that has acquired non susceptibility to atleast one agent in three or more categories of antimicrobials.^9^

The present study was conducted to detect the antibiotic susceptibility profile and prevalence of MDR P. aeruginosa isolated from different clinical samples, collected from different hospitals of Karachi.

## METHODS

This cross sectional study was conducted in Microbiology Department, University of Karachi, from January 2012 to January 2013. Clinical samples (urine, pus, wound swabs, ascitic and bronchial fluid, blood) were collected from different hospitals of Karachi.

***Inoculums for Antimicrobial Sensitivity Testing (AST):*** Overnight culture was further incubated on shaking water bath for 2 h, the turbidity of inocula was matched with 0.5 MacFarland standard suspension. McFarland standards were prepared by adding specific volumes of 1% Sulphuric acid and 1.175% Barium chloride. McFarland standard 0.5 contain 99.5ml of 1% Sulphuric acid and 0.5ml of 1.175% Barium chloride. The turbidity of standard was comparable to bacterial suspension containing 1.5 × 10^8 ^CFU/ml.

***Preparation of Agar Media:*** Agar plates were prepared using dehydrated media according to the instructions of the manufacturers. After autoclaving, media was allowed to cool down to ~45^0^C, then poured in the Petri plates. These Petri plates can be safely stored in refrigerator with proper precautions for about 2 weeks.

***Identification***: Clinical isolates were identified by standard methods. They were inoculated on Blood agar (Oxoid), Mac Conkeys agar (Oxoid) and Cystine lactose electrolyte deficient agar (Oxoid). Isolates were identified on the basis of colony morphology, Gram staining and biochemical tests including Catalase, Oxidase, Sulfide, Indole, Motility, Citrate, Urea, TSI reaction, Pyocinin production and Lactose

***Antimicrobial Testing:*** Antimicrobial activity was performed by using NCCLS standards.^10^ Modified Kirby-Bauer disc diffusion method was used for antimicrobial testing. Antibiotic disc of Piperacillin/ tazobactam (110µgm),Imepenem (10µgm), Meropenem (10µgm), Cefuroxime (30µgm), Cefipime (30µgm), Ceftazidine (30µgm), Amikacin (30µgm), Gentamicin(10µgm), Ciprofloxacin (5µgm), Nalidixic acid (30µgm), Co-trimoxazole(5µgm), were purchased from Oxoid.

Mueller Hinton plates were seeded with 0.5 MacFarland suspension matched turbidity inocula and antibiotic disc were placed on them. Results were interpreted after 24 hours of incubation at 37^0^C by measuring zones of inhibition around discs. Experiments were conducted in triplicate to authenticate the results.

Statistical analysis was performed by SPSS version 17. Frequency of MDR *P. aeroginusa *and percentage of resistant antibiotics were calculated.

## RESULTS

*P. aeroginusa* were isolated from clinical samples. Out of 100 isolates, 30 were found to be MDR *P.aeroginusa *(Table-I)*. *ATCC *P. aeruginosa* 27853 was used as positive control. 

*P. aeruginosa* was confirmed by negative sulphide and lactose tests and positive results in rest of biochemical identification tests. The maximum number of MDR *P. aeroginusa *were isolated from pus samples (33.3%), followed by wound swabs (26.6%), bronchial fluid (23.3%), urine (10%) and blood samples (6.6%), as represented in Table-I. The resistance patterns of MDR *P.aeruginosa* against antimicrobial agents are presented in Table-II. Highest resistance was observed against Cephalosporin group of antibiotics. While, more than 50% of isolates subjected to antimicrobial testing were found resistant to Piperacillin/Tazobactum. Around 40% of isolates were resistant against Carbapenems. While 90% of the isolates were sensitive to Amikacin.

**Table-I:** Distribution of MDR *P. aeruginosa* in Clinical Samples

***S. No***	***Samples***	***No. of Isolates***	***Percentage***
1	Pus	10	33.3
2	Wound swabs	8	26.6
3	Bronchial Fluid	7	23.3
4	Urine	3	10
5	Blood	2	6.6

**Table-II:** Antibiogram of MDR *P. aeruginosa*

***S. No***	***Antibiotics***	***No. of MDR P.aeroginosa Resistance***	***Percentage Resistance of MDR P.aeroginosa***
1	Piperacillin/Tazobactum	17	56.6
2	Imepenem	12	40
3	Meropenem	12	40
4	Cefuroxime	30	100
5	Cefixime	30	100
6	Ceftriaxone	26	86.6
7	Cefepime	23	76.6
8	Amikacin	3	10
9	Gentamicin	9	30
10	Ciprofloxacin	8	26.6
11	Nalidixic acid	8	25
12	Co-trimoxazole	6	20

## DISSCUSSION

Infections of multi drug resistant *P. aeruginosa* are increasing worldwide. It is an important pathogen frequently involved in various infections especially in severely or terminally ill patients.^11^^,^^12^

Altered target sites, bacterial efflux pumps, enzyme production or inhibition, loss of membrane protein, etc are different mechanisms mediated by multidrug-resistance (MDR) P.aeruginosa.^3^ This study revealed the susceptibility pattern of antibiotics used and the frequency of MDR *P. aeruginosa* found in the city of Karachi.

The present study showed a 30% frequency of MDR *P. aeruginosa*, while Gill et al, reported a 22.7% incidence in Islamabad.^13^ Another study was conducted in Peshawar in 2009 by Farhatullah et al, which reported 29% prevalence of MDR *P. aeruginosa*.^14^

Increasing resistance of beta-lactam in nosocomial *P. aeruginosa* has become a serious threat particularly against third and fourth generation Cephalosporins, is of major concern. There are a lot of molecular mechanisms to develop resistance against these antibiotics; generation of extended-spectrum beta-lactamases (ESBL), by incorporation of bla genes in integrons and inability of porin genes to enhance their expression level and/or alteration of antibiotic target sites.^15^

Present study showed that *P. aeruginosa* was found to be highly resistant against cephalosporin group of antibiotics. Study reported by Wang et al, explained the absolute resistance of Ampicillin, Cephazolin, Cefuroxime and Cefotaxime, which is in accordance with our results.^16^ Our study was also supported by Hamza et al, exhibited 100% resistance against Cefixime.^17^ While Jombo et al, reported 86% susceptibility of *P. aeruginosa* against cefurixime.^18^

Carbapenems the most significant group of antibiotics against MDR *P. aeruginosa* but the development of Carbapenems resistance is becoming a challenge for health care professionals and limited the therapeutic options. Sufficient measures are required to prevent the spread of Carbapenemase encoding gene to other bacteria.^19^ The current study demonstrated that 60% *P. aeruginosa* were resistant against Carbapenem antibiotics (Imepenem, Meropenem)*. *Rodríguez-Martínez JM et al, showed that 87% of strains of *P. aeruginosa* were resistant against Imepenem.^19^ Another study reported 100% resistance against Carbapenems,^20^ it is very obvious that efficacy of this particular antibiotic is declining. Clonal spread contributes lesser importance in the statistics and epidemiology of infections caused by P. aeruginosa, and the main mechanism associated with increased resistance to Imipenem was reduced expression of OprD (outer membrane protein) found in the isolates.^19^

Fluroquinolone compounds are one of the important antimicrobial agents that have been used for variety of infections. New groups of Fluroquinolone are beneficial against Gram-negative and Gram-positive bacteria as far as older Fluroquinolones are concerned, they were effective against aerobic Gram-negative bacteria.^21^ Present study showed 75% sensitivity against Ciprofloxacin and Nalidixic acid, while 100% resistance against Ciprofloxacin was exhibited in one study.^20^ Similarly, 87.8% resistance was also claimed by another study.^13^ Abdallah et al, reported 100% resistance to Nalidixic acid.^22^

Aminoglycosides is a significant member of broad spectrum antibiotics with a peculiar structure of an aminocyclitol ring. They are outstandingly active against aerobic and facultative aerobic Gram-negative bacteria. They mainly act by inhibiting protein synthesis and break cell membrane.^23^ The current study explored that anti *P. aeruginosa* effect of Amikacin was higher than Gentamicin. Amikacin was constructed as a weak candidate for the enzymes that are responsible to bring chemical modifications but some organisms have developed specific enzymes to inactivate Amikacin.^24^ One study declared 21% resistance against Aminoglycoside.^21^ Moreover, one more study explained 83% resistance to Amikacin. The resistance of clinical isolates to Aminoglycoside antibiotics varies with the specific drug, the microorganism, its mechanism of resistance, the geographic area and many other factors.^25^

Cotrimaxazole is the synergistic combination of Trimethoprin and Sulfomethaxazole. This study showed 20% resistance of *P.aeruginosa* against Cotrimaxazole but in contrary 100% resistance was documented in Libya^22^ as well as 47% resistance reported by study conducted in Nigeria.^18^

This study indicated Amikacin as an efficient treatment of choice against MDR *P. aeruginosa* among all the tested antibiotics.

## CONCLUSION

The emergence of MDR *P. aeruginosa* and its continual spread is out of debate. Antibacterial research is not sufficient to keep pace with the clinical challenges of MDR bacterial crises. Lack of new drug pipelines and other issues are leaving disastrous consequences on the health of community. To overcome such issues, new therapeutic agents with maximum efficacy, lesser toxicity and cost effective in nature are urgently needed. Epidemiological studies and strict laws regarding antibiotic policies should be constructed to limit the unnecessary use of antibiotics so that spread of multidrug resistance can be avoided.

## Authors contribution:

**Dr. Fouzia Khan **designed and performed the study

**Dr. Adnan Khan **did statistical analysis and editing of manuscript.

**Dr. Shahana Urooj Kazmi **supervised the study from each and every step starting from sampling, data collection, experimental work, results and writing of this manuscript.
